# Validity and reliability of the Greek version of Pregnancy Outcome Questionnaire (POQ)

**DOI:** 10.34763/jmotherandchild.20222601.d-22-00001

**Published:** 2022-07-20

**Authors:** Antigoni Sarantaki, Anastasia Nomikou, Athanasios Raptis

**Affiliations:** Department of Midwifery, University of West Attica, Athens, Greece; Freelance Midwife, MSc Athens, Greece; Department of Economics, National and Kapodistrian University of Athens, Athens, Greece

**Keywords:** Validity and reliability, Pregnancy Outcome Questionnaire (POQ), pregnancy-specific anxiety, pregnancy-related stress

## Abstract

**Background:**

During the last decades a considerable increase in biological and psychosocial approaches have occurred so as to enhance the study of prenatal period. This study aimed to investigate the validity and reliability of the Greek version of Pregnancy Outcome Questionnaire (POQ) in assessing pregnancy-related stress.

**Material and methods:**

The study group consisted of 135 first-time expectant women with good knowledge of the Greek language, low-risk pregnancies and a gestational age of ≥24 weeks. Questionnaires containing the POQ scale questions in addition to other questions and scaleswere distributed in printed and digital format at private gynecological clinics. The collected data were analysed using the SPSS software.

**Results:**

The POQ scale score showed satisfactory reliability (Cronbach’s alpha = 0.8), while the factor analysis showed a major factor with an eigenvalue of 4.17 and an overall interpreted factor variance of 41%. The sample showed moderate intensity values on the scale. We observed that pregnancy-related characteristics affected the scale, while no significant correlations with demographic variables were recorded.

**Conclusion:**

The results of the reliability and factor analyses evaluating the scale structure indicated that the tool performed well in Greek, had a compact structure with satisfying reliability, and is suitable for use in the Greek pregnant population. However, additional research is warranted to investigate the effect of various additional factors on the scale.

## Introduction

Pregnancy changes a woman's life prospects and may have significant implications for her health, well-being and social roles [1,2]. The stress rate experienced during pregnancy is higher than that of the general population. Pregnancy-related anxiety is a multidimensional type of anxiety; its definition varies from general anxiety and depression[2,3,4]. Furthermore, it affects both the mother and the child [5] and may occur in 14.4% of pregnant women [4].

The attributes of this type of stress relate to pregnancy-specific fears and concerns around the foetus’ well-being, childbirth per se, body and appearance issues, and puerperium [3,6].Bayrampour et al. [5] defined pregnancy stress as ‘nervousness and fear about the baby’s health, mother’s appearance and health, experience of the healthcare system, pregnancy-related socioeconomic factors, labour, and parenthood; all of which are accompanied by intense anxiety and physical symptoms’.

Significant evidence has gathered on the association between pregnancy-related stress and adverse birth outcomes with an increasing number of measures of this type of stress being developed worldwide. However, the introduction of these measures has not always been theoretically or psychometrically grounded, resulting in questions about the quality and direction of such research [7].

The Pregnancy Outcome Questionnaire (POQ) scale aims to assess the concerns of pregnant women regarding the outcome of their pregnancy. Four studies have used the POQ developed by Theut in 1988 [8], which was introduced specifically for the evaluation of anxiety in pregnancy subsequent to perinatal loss [8,9,10,11]. The scale comprises 15 questions addressed to gestating women, with or without a history of perinatal loss (Appendix). Moreover, it evaluates the intensity of pregnancy-specific and motherhood-related preoccupations of the expectant mothers. This study’s objective was to investigate the validity and reliability of the Greek version of the POQ in assessing pregnancy-related stress in women with or without, a history of perinatal loss.

## Material and methods

### Ethics

The study was approved by the Institutional Review Board. The participants were chosen according to their availability and their willingness to take part in the research, in order to avoid affecting the results by unwilling participation. Eligible women were approached by trained research assistants as they awaited care in the prenatal care facility centre. Interested women were provided information about the purpose and nature of the study. Those meeting eligibility requirements and willing to participate provided their signatures to indicate informed consent to complete the questionnaire and to allow access to their medical charts. To minimise participant burden, questionnaires were completed in a private room at the prenatal care facility while participants awaited scheduled appointments. Before answering the questionnaire, all participants received a consent form describing the purpose of the research, type and processing of data to be collected, and personal data protection policy according to Helsinki Declaration and its later amendments. Informed consent was given by all participants before completing the questionnaire. In the absence of informed consent, they were excluded from the study.

## Participants

We recruited pregnant women who were monitored in six private clinics and would mostly give birth in private maternity hospitals in Athens. The inclusion criteria were pregnant women, an age limit of≥18 years, first-time pregnancy after natural conception (preferably), good knowledge of the Greek language and a gestational age of ≥24 weeks. The criterion of primiparous subjects was applied taking into consideration that women who have already delivered a baby once, have the experience of transition into motherhood and, therefore, know what to expect from the process of childbirth and parenthood [12].Women may experience anxiety or fear over the upcoming changes, when becoming mothers for the first time [13]. Additionally, participants were asked to answer the questionnaire when reaching a minimum gestational age of 24 weeks, because from this week onward, extremely premature neonates demonstrate slowly increasing chances for survival [14]. The data were collected between July 2021 and October 2021. A total of 135 pregnant women responded to the questionnaire.

## Data collection

Questionnaires were distributed in printed and digital format using Google Documents. It has been shown that answering online surveys does not differ significantly from the traditional paper-and-pencil method [15]. Points of distribution were private obstetric/gynaecological clinics. Questionnaires were handed out by midwives only to gestating women/mothers-to-be whose current obstetric history indicated a low-risk pregnancy. The participants’ demographic characteristics and personal and reproductive history were collected at the beginning of the questionnaire using questions on age, marital status, education, family income and obstetric history associated with possible previous pregnancies (childbirths and miscarriages).

## Tools

The POQ scale was designed by Theut et al. [8] to examine the following hypothesis: in a pregnancy that follows a perinatal loss, parents experience pregnancy-specific stress rather than general stress. The construction methodology was based on interviews with couples expecting a child and having a history of perinatal loss [3], including miscarriage (early prenatal loss). The POQ scale addresses both first-time parents and those who already had a pregnancy in the past [8].

The POQ’s 15 questions are graded on a Likert scale from 1 to 4, where 1 represents ‘never’, 2 ‘occasionally’, 3 ‘often’, and 4 ‘almost always’. Some questions are reverse-scored. The scale scope range is 15–60 when treated as a sum and 1–4 when treated as an average. Regarding reliability, studies have reported Cronbach *α* reliability index values of 0.8. Regarding validity, the tool is successfully differentiated into anxiety values among pregnant women, with and without a history of perinatal loss, in terms of the degree of concern they express about their pregnancy outcome [16,17]. The POQ scale was translated and weighted in Greek, by Tsartsara Eirini [16].

## Statistical analysis

The data collected were appropriately coded and processed via SPSS v.24.0 software. Cronbach’s alpha index was calculated to assess the reliability of the study scales [18]. The t-test for two groups and the analysis of variance (ANOVA) for three or more groups were usedto test the statistically significant between-group differences of the scale means. Furthermore, to confirm the structure of the scale, the factor analysis method was implemented with the principal component analysis technique.

## Results

The subjects of the study aged between 22 and 50 years (mean = 33.2, standard deviation [SD] = 5.16), while the average gestational week at the time of answering the questionnaire was 29.40 weeks (SD = 4,78). The absolute and relative frequencies of demographic characteristics and pregnancy are shown in Tables 1 and 2, respectively.

**Table 1:** Frequencies and percentages of the demographics of the sample

Variable	Category	n(f%)
Education	High school / secondary education	31(23%)
	Tertiary education (University / TEI)	73(54.1)
	Master’s degree (completed or pursuing)	27(20%)
	Doctoral degree (completed or pursuing)	4(3%)
Marital Status	Single	16(11.9)
	Married	112(83%)
	Cohabitation agreement	6(4.4%)
	Other status	1(0.7%)
Do you have other children besides the current pregnancy?	No	135(100%)
	Yes	0(0%)
Salary	<500€	3(2.2%)
	500–1,000€	42(31.1%)
	1,000–1,500€	31(23%)
	1,500–2,000€	26(19.3%)
	2,000–3,000€	26(19.3%)
	>3,000€/month	5(3.7%)

Abbreviations: f, frequency; n, number; TEI, Technological Educational Institute

**Table 2:** Frequencies and percentages of pregnancy-related characteristics

Variable	Category	n(f%)
Your pregnancy is:	Singleton (onefoetus)	128(94.8%)
	Multiple (two or morefoetuses)	7(5.2%)
Has there been any abnormal condition in your pregnancy so far?	No	109(80.7%)
	Yes	26(19.3%)
The present pregnancy is a result of:	Natural conception	133(98.5%)
	Assisted reproduction	2(1.5%)
Have you had a miscarriage in the past?	No	104(77%)
	Yes	31(23%)
If you had a miscarriage in the past, this occurred more than once?	No	25(80.6%)
	Yes	6(19.4%)
Reason for not achieving conception so far?	Female factor	2(1.5%)
	Male factor	4(3%)
	No reason	129(95.5%)
How many times in total have you used an assisted reproduction method?	1	1(0.75%)
	2	1(0.75%)
	None	133(98.5%)
The present pregnancy is the result of which assisted reproduction method?	Ovulation induction	1(0.75%)
	Sperm insemination	1(0.75%)

Abbreviations: f, frequency; n, number

The reliability of the scale, measured using Cronbach’s alpha index, was 0.821, while removing the indicators ‘Cronbach if item deleted’ showed no improvement of the reliability index value in any question. Table 3 demonstrates the mean values and SD for the scale items. For the POQ scale in the whole sample, and for scale 1–4, we found: Mean = 2.10, SD = 0.42, Min = 1.13, Max = 3.33, Median = 2.06, Q1.3 = (1.80; 2.40) These values must be assessed under the assumption that the definition interval of the scale is [1,4]. The average value of POQ was 2.1, the median 2.06, and the SD 0.42. To evaluate this tool, we considered a four-point scale; the neutral intermediate value of the scale was 2.5. According to the definition of the scale, a higher value indicates a greater concern by the pregnant woman. Therefore, we concluded that on average the sample had low anxiety values.

**Table 3:** Frequencies, percentages, means and standard deviations for the POQ scale items

	n(f%)				
POQ Items	Never	Sometimes	Often	Almost always	Mean(SD)
POQ1(R)^1^	4(2.96%)	6(4.44%)	56(41.48%)	68(50.37%)	3.4(0.72)
POQ2	42(31.11%)	55(40.74%)	32(23.7%)	6(4.44%)	2.01(0.86)
POQ3	9(6.67%)	43(31.85%)	49(36.3%)	33(24.44%)	2.79(0.89)
POQ4	11(8.15%)	48(35.56%)	56(41.48%)	19(14.07%)	2.62(0.83)
POQ5	88(65.19%)	30(22.22%)	13(9.63%)	3(2.22%)	1.49(0.76)
POQ6(R)	0(0.00%)	13(9.63%)	49(36.3%)	72(53.33%)	3.44(0.67)
POQ7	24(17.78%)	62(45.93%)	43(31.85%)	5(3.7%)	2.22(0.78)
POQ8	56(41.48%)	55(40.74%)	19(14.07%)	4(2.96%)	1.78(0.8)
POQ9	72(53.33%)	46(34.07%)	13(9.63%)	4(2.96%)	1.62(0.78)
POQ10	47(34.81%)	52(38.52%)	30(22.22%)	5(3.7%)	1.95(0.85)
POQ11(R)	17(12.59%)	41(30.37%)	58(42.96%)	19(14.07%)	2.59(0.88)
POQ12	4(2.96%)	53(39.26%)	54(40%)	24(17.78%)	2.73(0.79)
POQ13(R)	2(1.48%)	8(5.93%)	54(40%)	71(52.59%)	3.44(0.68)
POQ14	60(44.44%)	43(31.85%)	25(18.52%)	7(5.19%)	1.84(0.9)
POQ15	1(0.74%)	20(14.81%)	49(36.3%)	65(48.15%)	3.32(0.75)

(R) indicates the questions for which reversal was required.

Abbreviations: POQ, Pregnancy Outcome Questionnaire; SD, standard deviation

ANOVA showed no correlation of the POQ with education (F_3;131;0.05_=0.241, p=0.867), marital status (F_3;131;0.05_=0.950, p=0.418), family income (F_5;127;0.05_=0.897, p=0.486), or miscarriage in the past (F_1;133;0.05_=0.099, p=0.754).

A statistically significant positive correlation was found between the POQ and the years of effort to conceive (Pearson’s r = 0.246, p = 0.037). A longer effort to conceive was associated with more intense anxiety about the outcome of the pregnancy. No correlation was found between the POQ scale and maternal or gestational age (p > 0.05).

**Figure 1:**
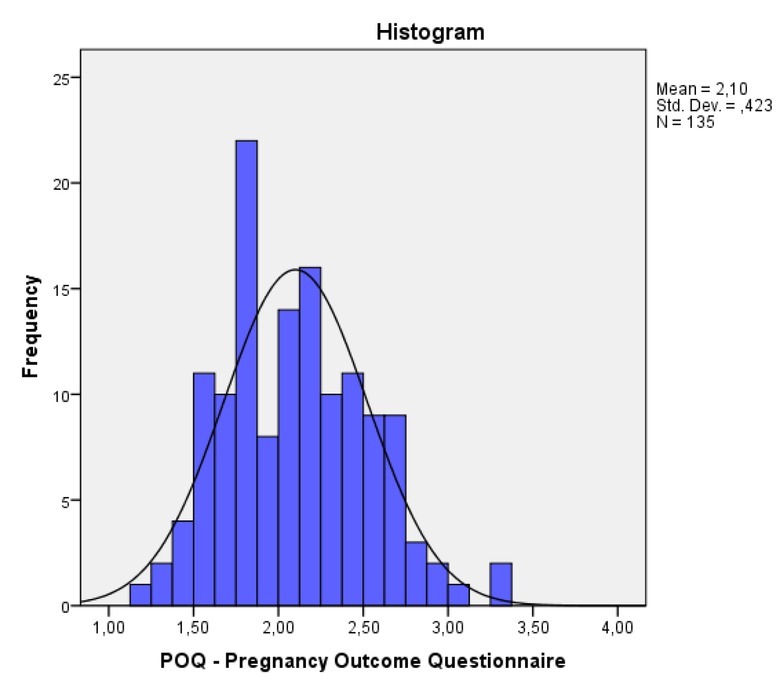
Distribution histogram of the POQ scale for total sample

**Figure 2:**
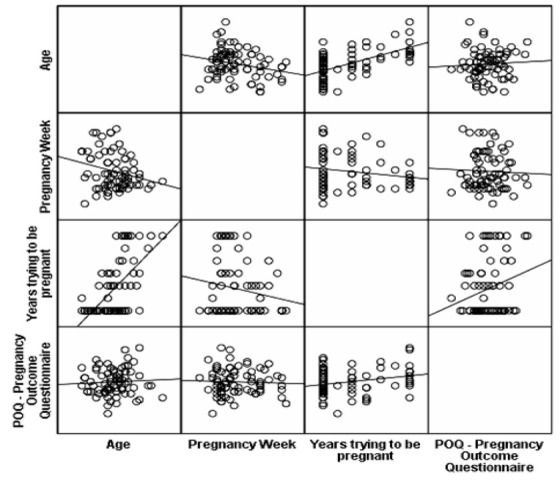
Multiple scatter dot matrix of the correlation between POQ scale and age, week of pregnancy, and years of efforts to conceive

The factor analysis showed a factor with an eigenvalue of 4.17, while the rest of the factors had eigenvalues marginally-to-well below the unit, with a total interpreted variance of 41%. The goodness-of-fit tests showed the following results: Kaiser-Meyer-Olkin Measure of Sampling Adequacy = 0.817; Approximate Chi-Square = 564,035; Bartlett's Test of Sphericity: df = 105; p <0.05. Furthermore, the negative signs of the reversed items were confirmed by the item loadings table.

## Discussion

Pregnancy outcomes depend on a complex network of interactions between the care provided, the individuals concerned and the context in which they occur [19]. Significant evidence has accrued on the association between pregnancy-specific stress and unfavourable birth outcomes with an increasing number of measures of pregnancy-specific stress being developed, worldwide [20]. High-quality value-based prenatal care can avoid or lead to timely recognition and treatment of maternal and foetal healthcare costs.

The objective of this work was to explore the validity and reliability of the Greek version of the POQ in assessing pregnancy-related stress. Few studies have examined the validity and reliability of the POQ scale, while no translations/translated version of the scale have ever been published.

In our research, demographic variables did not have an impact on the POQ scale. This result is in accordance with the findings of Tsartsara and Johnson [17] claiming that none of the examined demographic characteristics affect gestational stress.

It has been reported that POQ assesses pregnancy-specific stress in gestating women with a history of perinatal loss, and the way stress increases the occurrence of such a history in subsequent pregnancies. Conversely, most subjects of our study had no history of perinatal loss; as a result, we evaluated the POQ scale regardless of a history of miscarriage. Therefore, this tool may assess gestational stress in pregnant women regardless of their known obstetric history.

We found a statistically significant positive correlation between POQ and the years of effort to conceive. Particularly, the concern about the outcome of the pregnancy intensifies when the years of effort to conceive increase, irrespective of a possible history of perinatal loss. This result has not been reported by previous studies.

In contrast to other studies using POQ to assess gestational stress [9,10,17,21], we found no association with past miscarriage. This may be attributed to the fact that only 23% of our sample had a miscarriage in the past.

Unlike former studies, our sample size was large (135 women). Particularly, apart from the work by Hutti et al. [21] containing a larger sample than ours, all studies using POQ enrol a sample of <50 individuals [9,10,17].

Nevertheless, this study is faced with certain limitations. The sample, albeit larger than that examined in previous studies employing the POQ scale, was not random; it involved pregnant women who received prenatal care in private clinics and would give birth in a private hospital. It is impossible to know whether the women participating in the study were at greater risk of experiencing specific gestational stress compared to the general pregnant population in Greece. Additionally, we did not include many women whose impregnation was induced by assisted reproduction, although they are reported to have higher rates of gestational stress than naturally conceived pregnancies [22,23]. However, we believe that these limitations do not decrease the importance of the findings. Thus, the research provides sufficient data for the reliability and validity of POQ in Greek.

Pregnancy-related stress is a distinct type of stress. The key characteristics of prenatal stress are similar to those defined for anxiety disorders. It is critical to identify the signs, symptoms and diagnostic thresholds that warrant prenatal intervention and to build up efficient, helpful and valid screening tools and intervention strategies to be extensively used. The behavioural implications of pregnancy-related stress seem to serve as important indicators of the severity of the condition. Anxiety, depression and stress during pregnancy can adversely affect perinatal outcomes for mothers and children. Particularly, anxiety in pregnancy is strongly related with shorter gestation and has unfavourable implications for foetal neurodevelopment and child outcome. Identification of this unique form of stress (linked with many harmful effects) can provide the opportunity for early prenatal diagnosis and timely intervention, with the potential result of an optimal course of pregnancy.

The results of the reliability and factor analyses evaluating the scale structure demonstrated that the tool performed well in Greek, had a compact structure with satisfying reliability and is suitable for use in the Greek population.

Future studies should also explore pregnancy-specific stress in women following an assisted reproduction technology, for whom the years of trying to conceive are longer in most cases. Further research should (a) examine the validity of the POQ in other populations of different language and/or culture and settings, to ensure that the questionnaire is psychometrically sound, and (b) investigate its evaluative properties to determine its usefulness as a clinical outcome measure in maternity health services research.

## Conclusion

Research and screening of pregnancy-related stress is restrained by a dearth of reliable psychometric evidence on self-report anxiety measures used in perinatal populations. The aim of the authors in this study was to adapt the POQ developed abroad, into Greek for the evaluation of perceived stress during pregnancy. It was established that the Greek version of POQ was a valid and reliable measurement instrument. This scale can be used for the evaluation of perceived stress in Greek pregnant population. Nevertheless, certain aspects of pregnancy-specific stress measures may have definite cultural relevance and may not translate well across cultures. A comparison between physical and assisted conception with a suitable experimental plan is warranted and recommended to advance knowledge in this area of study, with a focus on outcomes that matter to women and their families.

## Figures and Tables

**Table:** 

*ITEM*	*TITLE*
POQ1 (R)	I feel confident that my pregnancy will proceed without any particular problems.
POQ2	I'm worried that nervousness about this pregnancy can affect its outcome.
POQ3	I'm worried about my new baby 's health.
POQ4	During this pregnancy I am intensely concerned about its outcome.
POQ5	I have been reluctant to talk to others about my pregnancy.
POQ6(R)	I feel that I can deal with the stress that this pregnancy causes me.
POQ7	I'm worried about whether my pregnancy will be normal or not.
POQ8	I'm afraid of being able to carry out this pregnancy.
POQ9	I feel overwhelmed by the anxieties and worries of this pregnancy.
POQ10	I'm worried about being able to take care of my new baby.
POQ11(R)	I feel convinced that I have some control over the outcome of this pregnancy.
POQ12	I am intensely concerned with the thought of childbirth and the birth of my baby.
POQ13(R)	I feel confident that my baby will be healthy.
POQ14	I am reluctant to make preparations for the arrival of the new baby (for example room, shopping, birth announcement).
POQ15	During this pregnancy I am extremely careful with issues, such as my weight, sex, smoking, physical activity, diet.
